# Comment on: Changes in the Sodium Content of New Zealand Processed Foods: 2003–2013 by D. Monro, C. Mhurchu, Y. Jiang, D. Gorton, H. Eyles. *Nutrients* 2015, *7*(6), 4054–4067; doi:10.3390/nu7064054

**DOI:** 10.3390/nu7075261

**Published:** 2015-07-17

**Authors:** Katherine Rich

**Affiliations:** Chief Executive, New Zealand Food & Grocery Council, 6146, New Zealand; E-Mail: Katherine.rich@fgc.org.nz; Tel./Fax: +64-4-472-7727

In this paper, the authors seek to measure changes in sodium levels in processed foods in New Zealand over the period 2003–2013. The purpose is to monitor progress in reducing such levels with the intent of lowering blood pressure. Such reductions and expected lowering is considered to be helpful in reducing cardio vascular and related health problems.

The research exercise involved collected data on sodium levels in nine categories of processed food product (323 products from four supermarkets in Dunedin) in 2003 and comparing those with levels found in the same categories (885 products from the “major” supermarkets in Auckland) in 2013. Analysis of these data was undertaken and conclusions drawn for sodium levels analysed as averages in the two time periods and for paired products at each of the two dates. The overall conclusion was that:

- Only very slow progress had been made in lowering sodium levels;
- Levels still fell far short of WHO recommended levels; and,
- A “renewed focus across the whole food sector” was needed if New Zealand was to meet its UN commitments.

The following comment shows that the difficulties of undertaking such research coupled with the research design and subsequent data interpretation mean that the conclusion is not justified, that significant changes are masked by the aggregation in the analyses presented, and that the research cannot defensibly be used to justify the calls it makes.

## Assumptions

Key assumptions made by Monro *et al.* are as follows:

- That processing which determines sodium level happens in New Zealand or is completely within the control of New Zealand manufacturers.

The limitation is that some products are imported and thus the assumption does not hold. That may well be a small proportion of total offerings, but, more difficult is the situation where intermediate inputs (ingredients mainly) are imported with sodium levels extant. In such cases reformulation is beyond the capability of local processors.

- That the presentation of products on supermarket shelves is an adequate proxy for their consumption.

This seems reasonable in that it reflects a market test in that consumers presumably buy and consume the products.

## Sample Size and Analysis Problems

As with category and product definitions, longitudinal studies create difficulties. Setting aside issues in comparing samples from supermarkets geographically remote from each other, the extent to which these two samples are representative of the population of processed food products presented on the two dates is questionable. It is unclear whether there are systematic differences between Auckland (in the north of New Zealand) and Dunedin (in the far south) for example. On the other hand, if we accept that “what is on the shelves” is a reasonable proxy for “what gets consumed” then the sampling method is likely acceptable, providing strident conclusions are not drawn from it, the limitations are recognised (especially the limitation that the sample is not likely representative of consumption patterns in any dependable fashion), and only limited reliance is made on results.

## Design and Method

Given the object of reducing sodium intake associated with processed food, measuring change requires a consideration of:

- Any change in the presence of sodium in the processed foods of interest; and,
- Any change in the consumption of such food over the time period in question.

Unless both factors are considered, any study will only produce a partial answer. Changes in volumes presented on the shelf are difficult to analyse (as the paper acknowledges) because the product mix alters, but volume consumed and change in volumes is as crucial as per unit presence of sodium, given changes observed in consumption patterns.

Given this requirement, a weighted average showing “how much of level ‘x’ made up ‘y’ percent of the offering” at each time period would tend to show quite different reductions in the offerings than simple raw means values indicate.

Complex effects and offsets, which the paper largely ignores, are at work. In the case of wheat for instance, mean per unit sodium levels appear to have increased from 297 to 317 but as a percentage of offerings this product has declined from 3% to 1%. The opposite effect also appears as described below.

## Comparing “Old with Old” not “Old with New”

A particular problem is that to examine change over time it is necessary to try to compare the products available for consumption in 2003 and their sodium content with an equivalent bundle consumed in 2013 AND to consider their relative contribution to the diet. The study did this by examining sodium presence in identical (or as close as is reasonably practical—an acceptable approach) pairs of products in 2003 and 2013. Overall, there appears to have been no statistical difference between sodium levels (at an acceptable level of confidence). However, no analysis of relative contribution to the diet was made.

The difficulty is that the bundle presented on shelves to customers in 2003 (323 products) is decidedly not the same bundle (885 products) presented in 2013, most probably due to product development, deletions, additions to ranges or ad hoc job lots imported to New Zealand. Conclusions which might hold for the 182 pairs cannot be neither generalised to today’s offering nor offer insights into the total change over time. For example, the number of canned tomato brands increases from 7 to a surprising 56 in 2013, some of which will have to be obscure tiny volume brands.

The 2013 bundle (885 products) is made up of the 323 products measured in 2003 plus an additional 562 products. The new 562 products may well be formulated with lesser levels of sodium. The study’s table one shows that more than 75% of the items examined exhibited a decline in sodium levels between 2003 and 2013. The development of new products, varieties and formulations mean that (by definition) pair-wise testing cannot be undertaken since not all products existed in 2003. If new products have lower levels of sodium—which may be the target of manufacturers in reducing sodium levels—then this would account for the 75% reduction but no apparent difference in pair-wise testing. Reformulation efforts may well have been primarily directed at new rather than existing products and (see below) the dominance of older products has declined.

As well, in relation to the choice of categories for review, if canned soup and frozen/canned meals had been selected, for example, there would have been far more significant reductions. The reformulation work in these categories is known to the authors.

## Changes in “Within Category” Mixes

Added to this problem is the potential understatement of sodium level declines occasioned by the fact that a given 2003 product in a given category may well, in 2013, make up a lesser proportion of all products in that category in 2013. Children’s cereals, for example, show not only a decline in mean per unit sodium levels from 574 (mg/100 g) to 358 (mg/100 g), but also a reduction from being an 11% share of the bundle to 5%. Every product shown in the breakfast bundle shows a decline and thus the full reduction in sodium consumed may well have fallen considerably more than the simple per unit comparison suggests.

Finally, there was no mention by the authors, again well known to them, that over 2003–2013 there have been many high profile initiatives championed by New Zealand companies to provide consumers with lower salt options. For example, there was a significant project that emerged in 2006 between Heinz Wattie’s and the Heart Foundation (through Pacific Heart Beat) to launch a reduced-fat, reduced-salt corned beef. It was a great product but it failed in the market and was gone in under a year because people just did not like it. It seems very remiss for this sort of example not to have been captured.

## Conclusions

Overall, this study creates the false impression that little has changed in 10 years, which is absolutely not the case.

Yours sincerely Katherine Rich Chief Executive New Zealand Food & Grocery Council

## References

[1] Monro D., Mhurchu C.N., Jiang Y., Gorton D., Eyles H. (2015). Changes in the sodium content of New Zealand processed foods: 2003–2013. Nutrients.

